# Evaluation of an Intervention to Improve Essential Obstetric and Newborn Care Access and Quality in Cotopaxi, Ecuador

**DOI:** 10.3389/fpubh.2016.00247

**Published:** 2016-11-21

**Authors:** Edward Broughton, Jorge Hermida, Kathleen Hill, Nancy Sloan, Mario Chavez, Daniel Gonzalez, Juana Maria Freire, Ximena Gudino

**Affiliations:** ^1^University Research Co., LLC, Bethesda, MD, USA; ^2^Johns Hopkins School of Public Health, Baltimore, MD, USA; ^3^Jhpiego, Washington, DC, USA; ^4^Independent Contractor, New York, NY, USA; ^5^Ministerio de Salud, Quito, Ecuador; ^6^Ministerio de Cultura, Quito, Ecuador; ^7^Universidad San Francisco de Quito, Quito, Ecuador

**Keywords:** essential obstetric and newborn care, quality of care, neonatal mortality, Ecuador

## Abstract

**Background:**

Despite improvements in health-care utilization, disadvantages persist among rural, less educated, and indigenous populations in Ecuador. The United States Agency for International Development-funded Cotopaxi Project created a provincial-level network of health services, including community agents to improve access, quality, and coordination of essential obstetric and newborn care. We evaluated changes in participating facilities compared to non-participating controls.

**Methods:**

The 21 poorest parishes (third-level administrative unit) in Cotopaxi were targeted from 2010 to 2013 for a collaborative health system performance improvement. The intervention included service reorganization, integration of traditional birth attendants (TBAs) with formal supervision, community outreach and education, and health worker technical training. Baseline (*n* = 462) and end-line (*n* = 412) household surveys assessed access, quality and use of care, and women’s knowledge and practices. TBAs’ knowledge and skills were assessed from simulations. Chart audits were used to assess facility obstetric and newborn care quality. Provincial government data were used for change in neonatal mortality between intervention and non-intervention parishes using weighted linear regression.

**Results:**

The percentage of women receiving a postnatal visit within first 2 days of delivery increased from 53 to 81 in the intervention group and from 70 to 90 in the comparison group (*p* ≤ 0.001). Postpartum/counseling on newborn care increased 18% in the intervention compared with 5% in the comparison group (*p* ≤ 0.001). The project increased community and facility care quality and improved mothers’ health knowledge. Intervention parishes experienced a nearly continual decline in newborn mortality between 2009 and 2012 compared with an increase in control parishes (*p* ≤ 0.001).

**Conclusion:**

The project established a comprehensive coordinated provincial-level network of health services and strengthened links between community, primary, and hospital health care. This improved access to, quality, use, and provision of essential obstetric and neonatal care and survival. Ecuador’s Ministry of Health is scaling up the model nationally.

## Introduction

In Ecuador, the institutional deliveries increased from 74% between 2000 and 2006 to 85% between 2007 and 2012. However, there remains lower use of maternal newborn services among the rural, poor, and indigenous population. A recent national maternal and child health survey showed that 37% of indigenous women did not receive four antenatal care (ANC) visits and only 30% had skilled delivery attendance in rural areas compared with 13 and 80% of Mestizo (non-indigenous) women, respectively. Only 15% compared with 38% received postpartum care, respectively (1). This disparity is partly attributable to a public health-care system that preferentially offered more skilled care to larger urban populations in provincial and county capitals. In 2009, the two largest public health providers, the Ministry of Health (MOH) and the Social Security Institute, offered ANC to the rural population through parish (sub-county) health centers with daytime but no night time, holiday, or weekend services. The majority of institutional deliveries are attended at district or provincial hospitals. Since most indigenous Ecuadoreans reside in rural parishes, difficult access to care and cultural preferences resulted in them receiving maternal and newborn health care from traditional birth attendants (TBAs), traditional healers, and family members.

Improving access and service quality from household to hospital across the continuum of antenatal to postpartum care is necessary to improve maternal and neonatal survival (2–5). The MOH’s quality improvement (QI) program has been committed to improving essential obstetric and newborn care (EONC) since 2003 (6). However, coordination with the private sector and community mobilization are critical to increase appropriate, timely access to essential care (7–9). To overcome geographic, transportation, financial, service readiness, and cultural gaps at the root of disparities in EONC access and use among rural, indigenous, and poor populations, the Center for Human Services (CHS) implemented a project in Cotopaxi from September 2009 to September 2013, supported by the Child Survival and Health Grants Program of the United States Agency for International Development (USAID).

Cotopaxi is one of Ecuador’s 24 provinces, located in the central highlands, and includes 7 counties and 33 rural parishes served by the MOH’s provincial referral hospital, 4 district hospitals and 20 parish health centers, 1 Social Security hospital, some ambulatory health centers, and 1 non-governmental organization (NGO) rural hospital. About 270 TBAs operating, there have historically been isolated from the formal health-care system. In 2008, the province had 384,500 inhabitants: 67% were rural, 28% were indigenous, and 90% were poor, with little access to or use of formal maternal newborn services (10). The project’s baseline survey found 49% of indigenous compared with 77% of Mestizo mothers made four or fewer ANC visits and 36% compared with 89% delivered in a health facility. At that time, there was little vertical coordination across the four health services levels (community care, parish ambulatory health centers, county hospitals, and the provincial hospital). There was also no horizontal coordination between public and private providers and other institutions which generally worked in isolation. This lack of coordination and the marginalization of TBAs were barriers to effective care with dysfunctional referral mechanisms, particularly for the poorer, indigenous, and rural population.

This study evaluated changes in facilities participating in the EONC network created by USAID’s Cotopaxi Project in terms of access and quality indicators of EONC and compared to non-participating facilities.

## Materials and Methods

The project’s aims were to improve access to and quality of EONC across the care continuum by creating a provincial-level network to coordinated maternal newborn health services and strengthened linkages between levels of care (Table 1). The project targeted 21 parishes with more than half the population in extreme poverty or more than 40% indigenous residents. The project was conducted collaboratively with national, regional, and local partners including the MOH, Social Security Institute, the Peasant Social Security Program, various NGOs, and local community organizations.

**Table 1:** **Cotopaxi project aims and interventions**.

Aim	Project intervention
Strengthen support for the EONC network	Engage local parish and municipal government representatives to promote awareness of and adherence to national laws mandating coordinated service provision through a public health-care system

Improve availability of EONC health services	Services in the provincial and five county hospitals were organized to ensure 24/7 availability of EONC Services were expanded by reorganizing provider work hours and by training and supervising all providers of obstetric and newborn care

Increase demand for EONC services	A weekly radio program was aired in Spanish and Kechwa. The radio program was about healthy behaviors, danger signs, and seeking professional care

Increase use and access of EONC services	Emergency community transportation committees were organized in several parishes The network formally integrated TBAs and community health workers (CHWs) to provide essential linkages with the community TBAs and CHWs were trained to identify maternal and newborn danger signs and risk factors and to refer them to health centers

Improve the quality of EONC care	MOH referral protocols were updated, and personnel were trained during a 4-day one-time training activity aimed at developing EONC knowledge and skills The network established and supported QI teams comprised of parish health center staff, CHWs, and TBAs who met monthly to work on improving access and quality of EONC services

### Intervention

The intervention commenced in 2011 in Pujilí county’s parishes and in Salcedo, Saquisilí, and Sigchos counties’ parishes in 2012. The network engaged previously isolated and competitive health services in a collaborative effort to improve service provision and utilize culturally sensitive, quality EONC, including referral and transportation of obstetric and newborn emergencies. In order to engage local parish and municipal government representatives to promote awareness of and adherence to national laws mandating coordinated service provision through a public health-care system, the project organized several meetings where such laws and national priorities were discussed. Emergency community transportation committees were organized in several parishes. These were responsible for notifying health facilities of patient referrals and requesting transport of emergency cases by ambulances which were present at all district/county hospitals in the province. Services in the provincial and five county hospitals were organized to ensure 24/7 availability of EONC for all women and newborns through labor, delivery, and the early postpartum period of highest risk for mother and newborn. MOH referral protocols were updated, and personnel were trained to use updated protocols during a 4-day one-time training activity aimed at developing EONC knowledge and skills. As it was not possible to add new staff, labor, delivery, and postpartum services were expanded by reorganizing provider work hours and by training and supervising all providers of obstetric and newborn care (residents, midwives, and nurses) in basic and comprehensive EONC.

The network formally integrated TBAs and community health workers (CHWs) with official MOH recognition to provide essential linkages with the community to increase access and use of EONC among the target population. QI methods were adapted for community-based implementation and used to assess and strengthen care and referral from all levels. The TBAs and CHWs were trained to identify maternal and newborn danger signs and risk factors and to refer them to health centers. Together with health center staff and community organizations, TBAs and CHWs created and continually updated community household maps that identified currently pregnant and postpartum women and newborns, including those with risk factors such as advanced maternal age, multi-parity, and prematurity. In each parish, TBAs and CHWs met monthly with health center staff to review the status of pregnant and postpartum women and newborns. They discussed the conditions of those at risk and how to address barriers including transportation, referral, and prompt care seeking. The network placed special emphasis on increasing coverage and quality of early postpartum/postnatal home visits within 48 h by TBAs and/or health center staff.

A weekly radio program prepared by a local communication team was aired in Spanish and Kechwa by seven radio stations that voluntarily contributed to the network. The radio program provided information about healthy behaviors, danger signs, and seeking professional care.

The network established and supported QI teams comprised of parish health center staff, CHWs, and TBAs who met monthly to work on common aims of improving access and quality of EONC services and to review and resolve problems. QI teams were supported at the provincial referral hospital and the five county hospitals. Hospital QI teams included doctors, nurses, midwives, and auxiliary nurses from maternity and newborn services. They met monthly to audit clinical records, calculate quality indicators, and implement rapid improvement cycles using the Plan-Do-Study-Act Model. This mechanism engaged supervision, training, and continual improvement in facilities with clinical, logistical, and administrative process deficiencies. A QI “managerial team” was organized at the provincial MOH office, with responsibility to collect and aggregate coverage and quality indicators reported by QI teams and use data to support health centers and hospitals to improve care.

### Evaluation

#### Household Survey

The Centro de Estudios de Población y Desarrollo Social, a local research organization, conducted baseline (April 2010) and end-line (July 2013) household surveys. These assessed the project’s effects in (1) access to and use of intra- and postpartum/postneonatal care, (2) women’s knowledge and practices, and (3) patient-reported quality of care. The survey questionnaire was adapted from the Knowledge, Practice, and Coverage Rapid Core Assessment Tool on Child Health (CATCH) 2008 (11); a USAID Health-Care Improvement Project household survey tool of mothers with children 0–23 months old (unpublished); and a Knowledge, Attitudes, and Practices Survey on maternal and neonatal health (12). The survey targeted mothers with a living child aged less than 24 months in Cotopaxi Province, excluding urban parishes of the capital, Latacunga. A multistage sample was selected. Primary sample units were the seven counties (second-level subdivisions) of Cotopaxi distributed proportionate to the counties’ population. Secondary sampling units were 30 parishes, proportionate to parish population. Parishes from the primary intervention county, Pujilí, were oversampled at baseline. After excluding census sectors with too few households to identify four or more children under 2 years old and replacing them with neighboring sectors, the number of sectors was selected by simple random sampling. All households in chosen sectors were visited to identify women with children under 24 months. Data were analyzed using difference-in-differences logistic regression controlling for potential confounders.

#### Facility Chart Reviews

Facility quality of care was assessed monthly through measurement of quality indicators calculated by facility QI teams using aggregate data extracted from clinical records’ reviews. Each month QI teams sampled clinical records by choosing 30 randomly from a list of all records of women who had received antenatal, delivery, and postpartum care in the month. When there were fewer than 30 records for 1 of the services in the month, all the records were chosen. Results were analyzed and used by facility QI teams to identify deficits and continuously improve care and were included in project monitoring reports. Data were analyzed using Pearson chi-square tests.

#### Simulations of Care by TBAs

Traditional birth attendants’ knowledge and skills for provision of early postpartum and postneonatal care were assessed by observing a sample of TBAs simulating care with an adult “mother actor” and a neonatal mannequin. Approximate every 3 or 4 months all the participating TBAs who had been trained to identify maternal and newborn danger signs and risk factors and to refer them to health centers, and who were participating in periodic meetings at health centers, were invited to a 3-day refresher training. At the start of each refresher training, their knowledge and skills level were assessed. Data were extracted and analyzed using Pearson or Fisher’s exact two-tailed chi-square tests comparing the first (pre) and last (post) trimester or the first and last measured performance weighted by the number of observations. Provincial government data provided by the Instituto Nacional de Estadística y Censos (INEC or National Institute of Statistics and Census) were used to estimate the proportion of deliveries for each month thus producing month-specific denominators. Change in neonatal mortality between intervention and non-intervention parishes in Cotopaxi provided by the INEC was analyzed using linear regression weighted by the counties’ number of annual births.

Comparisons of intervention and non-intervention parishes limited to the rural sample are presented to show changes in access and use of care and counseling as they include the project population and similar rural, neighboring parishes that constitute the comparison group. We analyzed the rural sample parishes for access and use of care considering that the integration of TBAs and CHWs to the formal network of services, an interventions aimed at increasing access and use of services, was implemented at rural parishes. The comparison group was made of geographical neighboring rural parishes with similar populations. The rural sample included 55% of the total household sample. With a rural baseline sample of 259 women and end-line sample of 237 women, the rural sample had over 99% power to detect an increase of 50% in postpartum visits within 48 h of birth in the intervention group compared with a 30% increase in the control group, given their respective baseline levels of 52.5 and 70.0%, with a Type I error of 0.05. A baseline to end-line comparison of the total sample was analyzed to assess change in women’s knowledge and practices, as neighboring non-intervention parishes were equally exposed to the project social and behavioral change media campaign. TBA quality of care was assessed quarterly by simulated case observation. Data were analyzed using Pearson’s Chi-square tests for proportions.

The study was approved by the CHS Institutional Review Board.^1^

## Results

The end-line intervention sample was more indigenous (42 versus 59%), and more women’s main occupation was farming their own land (64 versus 31%, both *p* ≤ 0.001, Table 2). Across both intervention and control groups, more end line (98%) than baseline (91%) survey participants had no or only primary education (*p* = 0.002) in the rural subsample.

**Table 2:** **Household survey for baseline and end-line sociodemographic characteristics of intervention and non-intervention parishes**.

		Non-intervention				Intervention				Δ Total
		Baseline		End line		Baseline		End line		
		%	*n*	%	*n*	%	*n*	%	*n*	*p*
Age group (years)	15–19	18	33	18	31	15	43	12	28	0.77
	20–29	57	103	57	99	44	124	48	115	
	30–39	22	40	23	40	32	89	32	75	
	≥40	3	6	2	4	9	24	8	20	

Education	None/primary	45	82	42	74	64	179	63	150	0.61
	Secondary	42	77	47	81	31	86	32	77	
	College	13	23	11	19	5	15	5	11	

Ethnicity	White	6.0	11	0	0	6	16	0.4	1	≤0.001
	Mestiza	84	152	81	141	50	140	40	96	
	Indigenous	5	10	16	28	42	117	59	140	
	Other	5	9	3	5	2	7	0	1	

Profession	House-wife	65	118	46	80	45	127	18	43	≤0.001
	Agriculture with own property	7	13	6	11	31	88	64	152	
	Other	28	51	48	83	23	65	18	43	

Marital status	Single	21	39	17	29	18	50	15	36	0.13
	Married	40	73	59	102	64	178	63	149	
	Live together	35	64	23	40	16	44	21	50	
	Other	3	6	2	3	3	8	1	3	

### Quality of Care

More women reported receiving counseling on newborn care in the intervention group than the comparison group (baseline 8%, end line 26%; *p* ≤ 0.001) (Table 3). Similarly, more postpartum intervention than comparison group women reported being counseled on newborn danger signs (intervention: baseline 12%, end line 23%; comparison: baseline 3%, end line 6%, relative increase *p* = 0.01) and family planning (intervention: baseline 6%, end line 18%; comparison: baseline 3%, end line 4%, relative increase *p* = 0.01) during home visits. There was no relative change in pregnant women reporting counseling on birth preparedness or danger signs of pregnancy during home visits or in women reporting counseling on breastfeeding, nutrition, or postpartum danger signs during home visits. There was no relative change in mothers reporting counseling on best practices and danger signs in pregnancy or birth preparedness counseling in health facility visits.

**Table 3:** **Rural household survey for baseline and end line: access and use of care and postpartum counseling**.

Access and utilization of care	Non-intervention		Intervention		*p*-Value for differences
	Baseline (%)	End line (%)	Baseline (%)	End line (%)	
Institutional delivery	89	86	52	50	0.226
Postnatal visit within first 2 days of delivery	70	90	53	81	<0.001
Institutional deliveries report postpartum discharge >2 days	3	66	5	75	<0.001
Identified postpartum complications at home	15	14	6	12	0.228
Postpartum complications at home referred	0	0	10	15	0.62
Postpartum home visit, counseling on newborn care	1	6	8	26	<0.001
Postpartum home visit, counseling on newborn danger signs and treatment	3	6	12	23	0.011
Postpartum home visit, counseling on family planning	3	4	6	18	0.009

### Access to Care (Rural Household Survey Sample)

Postnatal visit within first 2 days of birth increased from 70 to 90% (a 29% increase relative to baseline) in the comparison group and from 52 to 81% (a 54% increase relative to baseline) in the intervention parishes (*p* ≤ 0.001) (Table 3). While the majority of rural comparison group deliveries occurred in facilities and only slightly more than half did in the rural intervention parishes, relatively more intervention group institutional deliveries received an institutional postpartum visit 2 days after delivery (*p* ≤ 0.001). Of postpartum complications identified at home and referred for higher level care, none presented in the comparison group, whereas 10% in the intervention group did so at baseline and 15% did at end line. This difference was not statistically significant due to the small number of complications.

### Women’s Knowledge, Home Best Practices, and Satisfaction (Baseline and End-Line Total Household Survey Sample)

At end line compared to baseline, more mothers could identify at least two routine newborn care best practices (97 versus 83%), newborn danger signs (30 versus 83%), and immediate drying of the newborn after delivery (31 versus 90%, all *p* ≤ 0.001, Table 4).

**Table 4:** **Household survey for baseline and end-line comparison of maternal knowledge between intervention and non-intervention sites**.

Knowledge survey items	Non-intervention		Intervention		*p*-Value for differences
	BL (%)	EL (%)	BL (%)	EL (%)	
Can identify 2+ routine newborn care best practices	85	89	83	97	<0.001
Newborn danger signs	21	31	30	83	<0.001
Immediately dry newborn after delivery	11	23	31	90	<0.001
Establish newborn’s skin-to-skin contact with mother	6	16	28	90	<0.001
Initiate breastfeeding within hour after delivery	26	32	35	90	<0.001
Umbilical cord care practice	4	4	15	89	<0.001
Frequent hand washing	27	13	22	91	<0.001

#### Facility Chart Audits

Facility quality measures (Table 5) showed consistent improvements in adherence with clinical standards over time for all but treatment of postpartum hemorrhage and newborn sepsis, in which there were few cases. During the first 3 months of intervention, 56% of health centers’ first antenatal visit sessions met clinical standards compared with 90% during the final 3 months% (*p* ≤ 0.001). At the first and last trimesters of intervention, 68 versus 98% of health center outpatient maternal postpartum visits met clinical standards; in hospitals, this increased from 0 to 81% (both *p* ≤ 0.001). In health centers, 73% during the first 3 months and 88% during the last 3 months of newborn facility ambulatory visits met clinical standards (*p* ≤ 0.001). Other facility inpatient indicators were only reported by area hospitals. At the first and last intervention trimesters, 68 versus 97% of facility deliveries received active management of the third stage of labor, and 13 versus 50% of facilities were fully compliant with inpatient immediate ENC standards (*p* ≤ 0.001). The difference in the number of first ANC visits and quality of care indicators reflects the large divergence in where and when people seek care. Most institutional postpartum and postnatal visits occurred in primary health centers, not hospitals.

**Table 5:** **Quality indicators from chart reviews: last compared with first trimester or year**.

Adherent with clinical standards	First trimester		Last trimester		*p*
	%	*n*	%	*n*	
Facility first ANC visits at rural subcenters	56	303	90	602	≤0.001
Ambulatory postpartum facility visits at rural subcenters	68	78	98	267	≤0.001
Ambulatory newborn facility visits at rural subcenters	73	77	88	170	≤0.001
Hospital deliveries with active management of the third stage of labor	68	57	97	147	≤0.001
Hospital administration of antenatal corticosteroids in premature births	67	3	93	27	0.16
Hospital management of premature rupture of membranes	0	1	100	2	NA
Hospital immediate essential newborn care	13	38	50	147	≤0.001
Hospital postpartum hemorrhage management	100	2	86	7	0.57
Hospital newborn sepsis case management	100	5	67	3	0.18

*Intervention parishes*.

#### Assessment of TBA Knowledge and Skills through Simulations

Assessment of TBA knowledge and skills show consistent improvements between the first and last project implementation trimesters (Table 6). As assessed by simulation observation, 2% of TBAs met standards of maternal postpartum physical examination in the first 3 months and 17% in the last 3 months assessed, and 54% of TBAs met standards of neonatal immediate postnatal physical examination in the first 3 months compared with 77% in the last 3 months assessed (Table 6, both *p* ≤ 0.001).

**Table 6:** **Assessment of TBA knowledge and skills based on observation of simulated postpartum exam**.

	First year		Last year		*p*	
	%	*n*	%	*n*		
TBAs can cite					
Two or more pregnancy danger signs	94	313	100	49	0.029
≥ Birth preparedness actions	82	320	98	49	0.004
Two or more postpartum danger signs	91	312	100	49	0.024
Two or more newborn danger signs	86	311	98	49	0.017
Two or more newborn best practices	91	312	100	49	0.024
TBA adherence with maternal postpartum physical examination standards (observation of simulation)	1	81	17	29	0.001
TBA adherence with newborn immediate postnatal physical examination standards (observation of simulation)	54	81	77	29	0.042

### Neonatal Mortality

The intervention group parishes’ newborn mortality rates declined from 5.5/1,000 live births to 4.2/1,000 live births between 2009 and 2012, compared with a slight increase from 4.5/1,000 live births to 4.7/1,000 live births in the control group (*p* ≤ 0.001).

## Discussion

This project dedicated about 2 years to establish a comprehensive, integrated provincial-level vertical and horizontal network that improved access to, quality, use, and provision of EONC for the most impoverished, indigenous counties of Cotopaxi, Ecuador. By supporting innovative, integrated, and community-oriented programing that aligned formal and informal health systems in the Province, the project transformed a multitude of isolated services and resources into a coordinated, cooperative entity. This appeared in line with reports of similar private–public partnerships in other resource-constrained settings (7–9). TBAs in Ecuador and other countries have been marginalized from the formal health system.

This study evaluated the intervention to improve birth attendant performance with the application of QI methods implemented in the EONC network. Our data support the value of formally recognizing and integrating TBAs into EONC services, consistent with observations that when integrated into a health-care system, TBAs can play an integral role in improving EONC services and maternal and newborn health (13–15). Moreover, through the creation of an EONC network, the project bridged profound gaps among fractured and isolated public and non-governmental institutions aligning formal and informal health systems and linking science-based health care with traditional and culturally responsive procedures. EONC was tailored to be implemented in a three-level network of services – parish health centers, district, and provincial hospitals – each one with its specific responsibilities and an effective referral system. QI methods were implemented at each level in the EONC network. Application of these principles resulted in an increase of access to and quality of EONC services. It greatly contributed to improving postpartum and neonatal care within the first 2 days of delivery, an outcome that likely contributed to neonatal survival improvement. While geographically and temporally limited randomized controlled trials have successfully mobilized communities to improve maternal and neonatal health (2, 4, 5), our project demonstrates impact for mothers and newborns at a time when outside support for health in Ecuador and Latin America was waning.

The Cotopaxi Project achieved its primary objective to increase the proportion of mothers and newborns receiving postpartum/postnatal care within 48 h of birth. The improvement was large considering the relative poverty and marginalization of the intervention group and the fact that the intervention was only fully implemented for 2 years in Pujilí and only 1 year in the other intervention parishes. This appears to be mostly attributable to improving postpartum visits in newborns delivered in institutions. A tradition of 40 days postpartum bed rest exists among the indigenous population of Cotopaxi, which may explain why there are many fewer ambulatory postpartum and postnatal than first ANC visits.

Improvement in the newborn care quality indicator was smaller than expected because 1 of the 11 criteria to completely adhere with newborn care norms, prophylaxis to prevent neonatal ocular infection, is unreliable and underestimates compliance because the norms recommended a specific brand of medication that was unavailable for many years. Suitable substitutes were used, but some clinics report compliance with this indicator literally (having not used the specified brand) while others did so figuratively (having used an effective alternative).

The differences in demographic characteristic and sampling may have underestimated the effects presented. Neither baseline nor end-line surveys were to include parish townships, yet both did, therefore to best represent the intervention area and improve the similarity of the groups, the analyses excluded parish townships. Reducing the sample size underestimated the statistical significance of some observed effects.

In this setting during the period of the intervention, there was no other project being implemented to attribute the changes seen apart from the one described here. We feel that the use of a control group accounted adequately for the secular trend of improvement that may have been occurring even in non-intervention sites.

While the project had little influence on the overall proportion of institutional deliveries, these results may simply reflect the fact that the end-line sample was distinctly more indigenous, poor, and vulnerable than the baseline sample. This contention is supported by the data. The Ecuadorean MOH has formally adopted the model with a dedicated budget and personnel to scale it up nationally. With the Ministry’s rollout, the EONC network model will continue to be adapted to the heterogeneous settings within Ecuador. Having been tested only in Ecuador, a middle-income country, the model should be adapted and evaluated in other settings, particularly in settings where significant disparities persist in maternal and newborn health outcomes and access to and quality of EONC services among disadvantaged populations.

## Conclusion

This USAID-funded project in Cotopaxi established a comprehensive, coordinated, and sustainable provincial-level network of health services and strengthened links between community, primary, and hospital health care. There was substantive improvement in access to, quality, use, and provision of essential obstetric and neonatal care and survival. The MOH in Ecuador is scaling up the model nationally.

## Author Contributions

JH and KH conceived the intervention and led in organizing its implementation; EB and NS conceived the evaluation, performed the analysis, and took the lead in writing the manuscript; MC, DG, JF, and XG assisted in implementing the evaluation, organizing and executing data collection, and provided input to all sections of the manuscript. All the authors approved the final version of the manuscript. EB and JH took primary responsibility for the responses to the reviewers’ comments.

## Conflict of Interest Statement

The authors declare that the research was conducted in the absence of any commercial or financial relationships that could be construed as a potential conflict of interest.

## Footnotes

^1^Center for Human Services/University Research Co., LLC 7200 Wisconsin Avenue, Bethesda, MD, USA.
